# Studies in Symptoms and Their Treatment.—I. Hæmatemesis

**Published:** 1905-05-06

**Authors:** C. O. Hawthorne

**Affiliations:** Physician to the Central London Ophthalmic Hospital, AssistantPhysician to the North-West London Hospital, and to the Royal Waterloo Hospital for Children and Women


### Hospital Clinics.

### STUDIES IN SYMPTOMS AND THEIR TREATMENT.—I. HÆMATEMESIS.

By C. O. Hawthorne, M.D., M.R.C.P., Physician to the Central London Ophthalmic Hospital, Assistant-Physician to the North-West London Hospital, and to the Royal Waterloo Hospital for Children and Women.

**Hæmatemesis** is a symptom which in many, perhaps in the majority of cases, is written in plain characters. That the blood is expelled from the stomach by an act of vomiting is obvious alike to the patient and the onlooker. In other instances, however, there may be doubt whether the case is one of hæmatemesis or hæmoptysis. The patient's judgment as between vomiting of blood in the former event and coughing up blood in the latter is often at fault and cannot be trusted. Further, in a free hæmoptysis (as also in an attack of epistaxis) some of the blood may be swallowed and subsequently vomited. On the other hand, a sudden hæmatemesis may produce cough, as a result, during the process of expulsion, of the entrance of some of the blood into the larynx. A quantity of bright red blood, more especially if frothy from admixture with air, clearly suggests hæmoptysis, whilst dark-coloured or brownish material, particularly if expelled with food materials, indicates hæmatemesis. Again, blood from the lungs will have its normal alkaline reaction, whilst from the stomach the reaction (unless the quantity of blood is large) may be rendered neutral or acid by admixture with the gastric juice. A point which may be helpful in investigating the history of a doubtful case is the condition of any sputa expelled after the occurrence of the hæmorrhage. In true hæmoptysis these continue, usually for several hours or days, to be stained with blood. Obviously this will not be the case with hæmatemesis, where, on the other hand, altered blood may be expected in the stools, giving to these a characteristic tarry appearance (melæna). The absence, therefore, of a history of blood-stained sputa following a free hæmorrhage is decidedly against a diagnosis of hæmoptysis. It is, however, consistent with bleeding from the gums, the pharnyx, or from adenoid growths. Hence such a history does not prove that a hæmorrhage is of gastric origin, though it strongly opposes a theory of bleeding from the lower respiratory passages. Another contrast between hæmatemesis and hæmoptysis is afforded by the fact that bleeding into the stomach much more usually causes the patient a sensation of faintness than does hæmorrhage from the lungs. Doubtless, however, in many instances there is no room for debate between these two possibilities. But cases occur in which the issue thus presented can only be decided on a careful scrutiny of all the facts, and the diagnosis is then directed by the considerations just discussed.

The chief **causes of hæmatemesis** are well known, and their several relationships to the hæmorrhage are fairly obvious. In addition, there are certain less familiar circumstances in which blood may be vomited, and where the event is not easily explained.

First among the more frequent conditions which lead to hæmatemesis must be placed ulceration—simple or malignant—of the gastric mucous membrane, and with this also, perhaps, of the other layers of the stomach wall. The bleeding is a necessary consequence of the extension of the necrotic process to the walls of larger or smaller blood-vessels. As a matter of clinical experience it is found that when a very free hæmorrhage occurs and the patient vomits a considerable quantity of bright red blood, the occurrence is usually due to a simple ulcer. Malignant ulceration, on the contrary, commonly produces more continuous oozing or repeated small hæmorrhages. In such circumstances the blood is usually retained in the stomach for longer or shorter periods, and the oxyhæmoglobin being converted by the gastric juice into hæmatin the vomit has the so-called "coffee-grounds" character. To this general rule, however, there are exceptions. Thus "coffee-grounds" vomit is often met with in simple ulcer when the hæmorrhage is relatively small in amount, and a very vascular malignant growth may readily, as a result of ulceration, be responsible for an abundant hæmorrhage. In some cases of ulcer of the duodenum blood may pass into the stomach with resulting hæmatemesis. But this does not usually occur, and hence in a balanced diagnosis between gastric and duodenal ulcer the fact of hæmatemesis would favour the former supposition. Either one or the other condition may, of course, be responsible for melæna. It may be well to mention, too, that in some cases of gastric ulcer there is neither hæmatemesis nor melæna; in others, again, hæmatemesis occurs without melæna, whilst in a third group melæna is the only evidence of hæmorrhage. Violent vomiting or retching by rupturing superficial vessels may cause the presence of blood in the vomit; the blood is usually seen as bright red streaks.

A second cause of hæmatemesis is portal obstruction, leading to passive congestion of the gastric mucous membrane. Such a result is particularly likely to occur in the direct and marked obstruction caused by cirrhosis of the liver. But cardiac valvular disease, and more especially mitral disease, may also produce congestion in the portal circuit as part of a general venous engorgement. Such congestion may partially relieve itself by oozing or small hæmorrhages from the surface of the mucous membrane. Hence both in hepatic cirrhosis and in mitral disease "coffee-grounds" vomit may occur. But in the former it is by no means uncommon for the patient to vomit more or less suddenly large quantities—even one or more pints—of dark venous blood and this, indeed, may be the first event of which he takes serious note. The explanation is that the free bleeding depends on the rupture of dilated veins in the lower part of the œsophagus and at the cardiac orifice of the stomach. In these situations there exists an anastomosis between the portal and systemic venous systems. Cirrhosis of the liver, by obstructing the portal return, leads to the passage of a larger volume of blood by this route. Consequently there is distension of the veins in the lower œsophagus, attended sometimes with ulceration of the mucous membrane, and should one or more of these varices rupture, blood may escape in large quantities. In a similar fashion cirrhosis of the liver may cause hæmorrhoids. Difficulty in return of the blood by the portal route is attended by opening out of the anastomosis between the inferior mesenteric and hæmorrhoidal veins, and should these dilated veins (hæmorrhoids) rupture, much blood may be lost per rectum. Possibly varices may exist in the gastric mucous membrane apart from hepatic disease and may be responsible for certain obscure cases of hæmatemesis.

The active congestion of the stomach wall produced by irritant poisoning must also be mentioned among the causes of hæmatemesis. The history of the case here usually decides the issue. Arsenic and perchloride of mercury may be mentioned as irritant poisons which frequently give rise to hæmatemesis.

An infrequent but well-recognised cause of hæmatemesis is the depreciated quality of the blood met with in such conditions as splenic anæmia, scurvy, purpura hæmorrhagica, yellow fever, acute yellow atrophy of the liver and phosphorus poisoning. Hæmatemesis and melæna have sometimes occurred in infants as the first symptoms of scurvy-rickets. There are also other conditions in infancy which lead to loss of blood from the alimentary canal; they will be discussed in connection with melæna.

Another unusual association with hæmatemesis is uræmia. The patients have for the most part been young women, and gastric pain and vomiting were included in the symptoms. Hence the suggestion of gastric ulcer was very direct, yet no ulceration was present on post-mortem examination. The obvious lesson is that the urine should be carefully examined before concluding that hæmatemesis means an ulcer of the stomach. Even the absence of albumen on a single occasion is not sufficient to negative a diagnosis of uræmia, as in such cases albuminuria is not always continuous.

In the gastric crises which sometimes accompany tabes dorsalis there may occasionally be either a "coffee-grounds" vomit or even a more free hæmatemesis. Here, again, pain and vomiting may appear to be specially related to the taking of food, and thus the diagnosis is apt to be confused. Especially is this the case when, as sometimes happens, ataxia and other conspicuous evidences of the disease are absent. Obviously, therefore, it is wise to remember that tabes dorsalis may be resposible for the vomiting of blood, and, with this in view, to examinne the pupils and test the knee-jerks, more especially, perhaps, if the patient is a man in middle life.

As a very rare event among the causes of hæmatemesis may be mentioned the rupture, with fatal consequences, of an aneurism into the stomach. In some instances the aneurism has affected one of the branches of the gastric arteries, in others it has arisen from the cœliac axis or the superior mesenteric artery.

Cases have occasionally been reported in which hysterical girls have swallowed blood obtained from the gums or pharynx and have afterwards vomited it. There are also examples of hæmatemesis which appear to suggest that loss of blood by the stomach depends on amenorrhœa; that it may, in short, be a form of vicarious menstruation. In this connection may be noted the occurrence of hæmatemesis at or about the menopause. Some of these cases are undoubtedly due to gastric ulcer. But there are others over which a doubt hangs. Certainly many women who complain of various disturbances at this epoch have occasional attacks of hæmatemesis with little or no other suggestion of the presence of ulcer of the stomach. And possibly the hæmorrhage may be the result of the circulatory and vaso-motor instability associated with the various changes which attend the menopause.

Lastly, there remains a group of cases in which hæmatemesis may be extreme, and may even prove fatal, without gastric ulceration or any of the other recognised causes of bleeding from the stomach. In some of these there have been found abrasions of the mucous membrane described by Dieulafoy under the term *exulceratio simplex*, but whether these are the cause or a consequence of the hæmorrhage remains uncertain. More recently there have been observed in connection with these abrasions minute "pore-like" openings leading into relatively large arteries, and the suggestion has been made that such a lesion is the initial stage of the ordinary gastric ulcer. It seems probable that in a certain proportion of the cases diagnosed as gastric ulcer and characterised by pain after food, vomiting and hæmatemesis, there is no ulcer in the ordinary sense, and it may be that in these cases the lesion is similar to that just mentioned. Certainly in some chlorotic girls with more or less marked dyspeptic symptoms, there occur attacks of hæmatemesis unattended by the local tenderness which is one of the recognised signs of gastric ulcer. The patient usually recovers rapidly from the effects of the hæmorrhage, and even though it recurs on one or more occasions, she does not suffer from the severe dyspepsia and loss of flesh which usually accompany ulcer of the stomach in its commonly admitted form. It may reasonably be doubted whether in these cases gastric ulcer is the correct diagnosis. And, pointing to the same conclusion, is the fact that whilst hæmatemesis is more common in females than in males, post-mortem records show that gastric ulceration is more frequently met with in the male sex. The practical importance of this aspect of hæmatemesis is that the loss of blood may appear to justify an operation and yet the surgeon may be unable to identify and secure the bleeding point. Hæmatemesis without appreciable lesion in the stomach sometimes also follows operations on the abdominal viscera.

To **test for blood in the vomit** the microscope is not reliable, as red corpuscles may not be found in blood altered by the gastric juice. Nor can the guaiacum and ozonic ether test be trusted. Teichmann's method is the most convenient and satisfactory. Mix some of the deposit, with a small quantity of common salt. Spread a little of the mixture on a glass slide and evaporate to dryness.

Place over a part of the residue a cover-glass, and allow some glacial acetic acid to flow under this. Gently heat (not boil) in the flame of a spirit-lamp for several minutes, adding more acid round the cover-glass to prevent undue evaporation. After cooling, a high power of the microscope will, if blood is present, detect the small brown rhombic crystals of *Hæmatin.*

The principles which govern the **treatment** of hæmatemesis are those which apply to the treatment of hæmorrhage in general. Rest and other measures which promote the plugging of the ruptured vessels with blood-clot must be adopted; whilst, if the bleeding has been so severe as to threaten syncope, means must be taken to sustain the heart's action. In this latter task it must be remembered that anything which acts violently or suddenly on the circulation may cause displacement of the clot and renewal of the bleeding.

In all cases the first claim is rest in the recumbent posture, and this should be maintained for several days; for even though a hæmorrhage is of only moderate amount on the first occasion its return, should this occur, may be on a severe or even a dangerous scale. Quiet and rest for a few days, combined with rectal feeding and the sucking of particles of ice, form a sufficient scheme of treatment for slight or moderately severe cases.

In some extreme cases there is danger that the patient may die of syncope. Hence growing pallor, a feeble and increasingly rapid pulse, and other signs of collapse, call for cardiac stimulants. Ammonia applied to the nostrils, ether (30 minims) by the hypodermic needle, or brandy per rectum are useful, and of course the patient is to be kept in the recumbent position and with the head at a rather lower level than the trunk. The introduction of saline fluid (ʒj common salt to a pint of boiled water) into the circulation is one of the most effective means of meeting the conditions established by severe hæmorrhage. In an emergency it may suffice to administer two or more pints of such fluid by slowly introducing the solution into the rectum. But subcutaneous injection is the better method. This may be effected through a hollow needle connected by indiarubber tubing with a glass funnel. The needle can be inserted into the inner aspect of the thigh, or at the axilla or abdominal wall. By raising the funnel one to two feet above the level of the bed a quart or more of the fluid can be gradually introduced and will be absorbed. Other measures necessary in severe cases are rectal feeding, nothing being given by the mouth unless it be small pieces of ice; cloths wrung out of iced water or ice itself applied to the abdomen, and some advise mustard packs to the lower limbs. Of medicines used to stop gastric hæmorrhage may be noted adrenalin chloride solution (15 to 30 min.), tincture of perchloride of iron (30 min.) mixed with an equal volume of glycerine, tannic or gallic acid (10 to 20 grains), and lead acetate (2 to 4 grains) in water, with a drop or two of acetic acid and solution of morphine hydrochloride (10 to 30 min.). Alum, sulphuric acid, and turpentine are also recommended. But all medication by the mouth should take the simplest possible form, in order to reduce the risk of vomiting, as this means the re-opening of the ruptured blood-vessels and the renewal of the hæmorrhage. Injections of hot water (112° to 116° F.) carried high up the bowel sometimes succeed in checking hæmatemesis. If, as occasionally happens, there is much restlessness, morphine by the rectum or hypodermically is indicated. To prevent further bleeding the policy of general and local rest must be continued for a time, and calcium chloride, which increases the coagulability of the blood, may be given per rectum in 30 to 60 gr. doses twice daily. If the case is one of hepatic cirrhosis, cessation of the bleeding should be followed by calomel and saline purgatives to relieve portal congestion. Saline purgatives, *e.g.* magnesium sulphate, with iron sulphate and strychnine are also useful after attacks of hæmatemesis in chlorotic girls. In exceptional cases dangerous hæmatemesis has been successfully treated by ligature of the bleeding vessel. But such a measure is rarely necessary, and as already indicated, the proposal to undertake it, unless the diagnosis of gastric ulcer is quite secure, is qualified by the risk that there may be no lesion in the stomach capable of recognition and treatment.

